# The role of stress-reactivity, stress-recovery and risky decision-making in psychosocial stress-induced alcohol consumption in social drinkers

**DOI:** 10.1007/s00213-018-5027-0

**Published:** 2018-09-12

**Authors:** James M. Clay, Matthew O. Parker

**Affiliations:** Brain and Behaviour Lab, School of Pharmacy and Biomedical Science, University of Portsmouth, Old St Michael’s Building, Portsmouth, PO1 2DT UK

**Keywords:** Alcohol, Impulsivity, Iowa gambling task, Risk-taking, Social drinking, Addiction, Substance abuse, Stress, Endophenotypes, Incentive sensitization

## Abstract

**Rationale:**

Chronic alcohol misuse can escalate into alcohol use disorder (AUD). The causal mechanisms through which recreational social drinking develops into compulsive uncontrolled alcohol misuse are multifaceted. For example, stress is an important risk factor that influences alcohol craving in both healthy and addicted individuals. In addition, those that are high in impulsivity/risk taking drink more and are at greater risk of developing addiction. At present, however, it is not possible accurately to predict those at risk of escalation in alcohol use, or of developing AUD.

**Objectives:**

The aim of this study was to investigate how underlying physiological and personality traits affect stress-induced craving for, and consumption of, alcohol, in a sample of social drinkers. The primary hypothesis was that impulsivity/risk-taking would modulate stress-induced alcohol craving and consumption.

**Methods:**

Thirty-nine participants (22 male and 17 female; mean age = 23.92 years [SD = 4.90]) were randomly allocated to ‘stress’ and ‘no-stress’ groups; in the stress group, participants took part in the Trier Social Stress Test (TSST). Participants completed several questionnaires and computer tasks in order to assess prior alcohol use, impulsivity/risk-taking, stress-reactivity, craving and physiological biomarkers of stress. Finally, participants completed a voluntary drinking task, in which increasing numbers of presses on a computer keyboard were reinforced with 5-ml shots of 37% ABV vodka (plus mixer).

**Results:**

Participants exposed to the TSST showed an increase in craving following the stressor. Several factors predicted voluntary drinking, including risky decision making, slow HR recovery from stress, poor vagal tone during recovery from stress and greater stress reactivity. Surprisingly, we found no correlation between craving and consumption.

**Conclusions:**

Our data suggest that variation in physiological stress parameters and poor decision-making abilities increase risk of stress-induced alcohol consumption. This may provide a useful translational framework through which we can further study early predictive markers for the shift between controlled recreational drinking to uncontrolled alcohol misuse, including AUD.

**Electronic supplementary material:**

The online version of this article (10.1007/s00213-018-5027-0) contains supplementary material, which is available to authorized users.

## Introduction

Alcohol misuse is one of the leading avoidable risk factors for morbidity and mortality, and presents a significant global health challenge (Rehm et al. 2009). In the UK, for example, alcohol misuse is estimated to cost £3.5 billion per year to treat and manage (Williams et al. 2014). In some individuals, alcohol misuse can escalate into alcohol use disorder (AUD) (Skinner and Allen 1982). AUD and other substance use disorders (SUDs) have traditionally been described as diseases (i.e. the Brain Disease Model of Addiction [BDMA]; e.g. Volkow et al. 2016), in other words, as a chronic, relapsing disorders, characterised by withdrawal symptoms in the abstinence of alcohol, compulsive alcohol seeking, anhedonia and social/familial problems (American Psychiatric Association 2013). More recently, however, groups such as the Addiction Theory Network (ATN; Heather et al. 2018) have begun to question the BDMA and instead describe addiction as a ‘disorder of choice’ (e.g. Heather 2017; Heyman 2009). Regardless of which theory you subscribe to, the consequences of addiction are severe and often lead to adverse health outcomes. It has been postulated that 20% of people will meet the clinical characteristics for diagnosis with AUD in their lifetime (Hasin et al. 2007; Teesson et al. 2010). Considering only half the world’s population consume alcohol, this statistic is all the more alarming (WHO 2014). Despite the substantial current and projected prevalence of AUD, and decades of research into alcohol misuse and AUD, we are still unable to provide effective treatment programs for those affected, or put into place effective protective factors to predict and prevent AUD from developing in at-risk individuals (Harris and Koob 2017). Current treatments for AUD are inefficacious, and in order to find curative or preventative treatments or interventions we must identify and understand the risk factors which cause both the acquisition and maintenance of the disease.

One previously identified and well-established risk factor for alcohol misuse is psychological stress. Chronic alcohol use causes neuroadaptations in stress pathways, for example within the hypothalamic pituitary adrenocortical (HPA) and sympathetic adrenomedullary (SAM) axes (Sinha 2012). These adaptations have been identified by cortisol response dysregulation (Kreek and Koob 1998) and deficits in emotional regulation (Sinha 2001). As a result of these neuroadaptations, AUD patients commonly present with an increased stress-induced craving for alcohol. Nevertheless, it is still difficult to predict the latency to and the likelihood of relapse between individuals due to inconsistent findings between patient groups (Sinha et al. 2011). In addition, how adaptations to the stress pathways affect the early development of AUD, or alcohol misuse more generally, is not well characterised.

Incentive sensitisation theory (Berridge and Robinson 2016; Robinson and Berridge 1993, 2000, 2008) posits that addiction (e.g. AUD) results from of a gradual shift in the intensity of the incentive motivation to consume alcohol. Specifically, the ‘incentive salience’ of alcohol-related cues become heightened within at-risk populations. In turn, this increases the ‘incentive motivation’ to drink. In other words, alcohol-related cues grab the attention of the individual, increasing their craving and thus increases their likelihood of drinking. When considered in the context of stress as an additional risk factor, the increased likelihood of drinking during stress may be related to an increased psychological or physiological arousal, thus increasing the incentive salience of alcohol-associated cues (Kreek and Koob 1998). A recent study (Salemink et al. 2015) found links between neuroticism and risk for alcohol misuse. Their findings suggest that neuroticism in adultescents tends to act as a protective factor until the point of alcohol consumption; in other words, alcohol reduces the anxiety levels in neurotic adultescents, which in turn increases the incentive sensitisation of this reduced state of anxiety, thus increasing their risk of alcohol misuse. Under Incentive Sensitisation theory, it could be predicted that in healthy individuals, (1) stress will increase craving for alcohol, (2) craving will be positively correlated with stress-reactivity (e.g. physiological arousal caused by the stress), (3) craving will be the strongest predictor of drinking and (4) liking will not be correlated with craving.

An alternative theory suggests, in predisposed individuals, that addiction arises following a ‘shift’ from controlled, voluntary substance use to a habitual, compulsive (addicted) state. According to this theory, compulsive drug taking develops as a result of an imbalance between goal-directed (mediated by the dorsomedial striatum [DMS]) and habitual behaviour (mediated by the dorsolateral striatum [DLS]) (Everitt and Robbins 2005). It is suggested that addicted individuals have a more dominant DLS-based ‘habit-forming’ neural circuitry, and this appears to be linked strongly to trait impulsivity (Everitt and Robbins 2005). It has been suggested that traits such as impulsivity represent sub-clinical ‘intermediate’ phenotypes (termed endophenotypes; Gottesman and Gould 2003) that are state-independent (i.e. present in at-risk individuals even when the disease is not). Trait impulsivity, defined as the propensity to proceed without forethought and take risks, despite potential adverse consequences, is one such neurocognitive endophenotypes. In humans, trait impulsivity is linked both to addiction and to relapse; addicts have higher levels of impulsivity, as do their (non-addicted) first-degree relatives (Bowden-Jones et al. 2005; Lawrence et al. 2009). Impulsivity has been causally, in pre-clinical studies, linked to compulsive drug-seeking in rodent models (Belin et al. 2009). Furthermore, previous research from our laboratory suggested that risk-taking (an individual tendency closely related to impulsivity) was positively correlated with stress-induced craving; i.e. people with greater risk-taking tended to have a larger alcohol craving post-stress (Clay et al. 2018). Interestingly, stress has been shown to modulate the shift between goal-directed and habitual responding, potentially as a result of stress activating the amygdala, which then influences the shift to habitual behaviour (Koob 2008). Under this theory, it could be predicted that, in healthy individuals, higher rates of stress-induced drinking will be observed in impulsive individuals.

There were two aims for this study. The first was to test the hypothesis that acute psychosocial stress (Trier Social Stress Test [TSST]; Kirschbaum et al. 1993) would increase voluntary alcohol consumption in a sample of social drinkers. The second aim of this study was to test how well variability in stress-induced voluntary alcohol consumption was predicted by incentive sensitisation theory or neurocognitive endophenotype theory. Based on neurocognitive endophenotype theory, it would be predicted that, even in non-addicted individuals, early signs of a loss of control over drinking (i.e. drinking more when feeling stressed) might be more common in impulsive individuals. However, under incentive sensitisation theory, there is no reason to suggest that impulsive individuals would drink more than others. Towards our second aim, we tested the hypothesis that, in a sample of social drinkers, impulsive/risk-taking individuals would consume more alcohol following exposure to stress.

## Methods

### Study design

This study employed a mixed design using both within-subject and between-subject independent variables (IVs). There was one within-subject IV with two levels (pre-intervention and post-intervention), and one between-subject IV with two levels (the intervention; either TSST or relaxation for 15 min). There were also several covariates, including alcohol-use measures and neurocognitive measures. The dependent variables were alcohol craving, quantified using as explicit craving (assessed via questionnaire), implicit craving (assessed via computer task) and number of drinks consumed on a voluntary drinking protocol.

### Participants

Thirty-nine participants were recruited from staff and students at the University of Portsmouth (22 male and 17 female; mean age = 23.92 years [SD = 4.90]) using opportunity sampling, i.e. through internal advertising through e-mail and by word of mouth. The advertising informed participants that we were interested in investigating what leaves some more at risk of misusing alcohol. Participants were also informed that they would take part in a mild stress test; however, specific details of the procedure were withheld from participants. To confirm the suitability of the participants, they were initially sent a pre-screening questionnaire via e-mail. Exclusion criteria included age < 18 or > 55 years, previously or currently undergoing treatment for alcoholism or treatment for anxiety, depression or any other stress-related disorder. To be sure this was the case, participants also completed the Patient Health Questionnaire for Depression and Anxiety (PHQ-4; Kroenke et al. 2009) as a secondary screening for depression and anxiety and the Alcohol Use Disorders Test (AUDIT; Bush et al. 1998) to screen for undiagnosed alcohol dependence. Any participant who score > 5 on the PHQ-4 or > 20 on the AUDIT was subsequently excluded. Owing to their effects on salivary cortisol (sC) and alpha-amylase (sAA) levels, there were several other exclusion criteria. Female participants could not be pregnant, breastfeeding or currently taking oestrogen- and progesterone-based contraception. For all participants, participants could not take any of the following medications within the past 24 h: barbiturates, phenytoin, carbamazepine, meprobamate, glutethimide, alpha-methyldopa, corticosteroids, non-steroidal anti-inflammatory agents (e.g. aspirin, ibuprofen]), codeine, propranolol, beta-adrenergic agonists, cyproheptadine and psychotropic medications (including sedative hypnotics). The study was approved in its current form by the University of Portsmouth Science Faculty Ethics Board (ref. SFEC 2016–068).

### Alcohol use and drinking behaviour

Alcohol use and drinking behaviour was evaluated using three measures: participants self-reported units of alcohol consumed per week, participants completed the AUDIT and participants completed the Binge Drinking Scale (BDS; Cranford et al. 2006).

Average alcohol use (units/week) was assessed through a single question ‘how many units do you typically consume in a week? Please note you cannot just count each drink as a unit of alcohol. The number of units depends on the different strength and size of each drink, so it can vary a lot. Here are some examples, single shot of spirits (25 ml, ABV 40%) = 1 unit, Alcopop (275 ml, 5.5%) = 1.5 units, small glass of wine (125 ml, ABV 12% = 1.5 units), large glass of wine (250 ml, ABV 12% = 3 units), bottle of beer/cider (330 ml, 5% ABV) = 1.7 units, can of beer/cider (440 ml, ABV 5.5%) = 2 units, pint of lower strength beer/cider (ABV 3.6%) = 2 units and pint of higher-strength beer/cider (5.2%) = 3 units’. The AUDIT, developed by the World Health Organisation (Babor et al. 2001) as a brief assessment of alcohol misuse for use in primary care and by researchers, was chosen to asses alcohol dependence. The AUDIT is scored on a scale of 0–40, where scores of > 20 would be considered dependent on alcohol, and > 30 severely dependent. The English version of the AUDIT has shown to have good psychometric properties for identifying alcohol dependence (Saunders et al. 1993; Stockwell et al. 1983). Additionally, the BDS was chosen to measure the level of binge drinking amongst the sample. Here participants are asked “What is the greatest number of drinks you have consumed in a 2-h period during the past 12 months?”. As the BDS originated in the US, Cranford et al. (2006) defined, binge drinking as consuming > 5 drinks for men or > 4 drinks for women on at least one occasion in the past 2 weeks. However, as the authors of this paper are based at a UK institution, we define binge drinking as consuming > 3 drinks in a single session, based on the NHS guidelines of binge drinking, i.e. consuming > 6 units in a single session (the mean unit/drink in the UK is 2).

### Neurocognitive measures

Impulsivity, risk-taking, sensation seeking and decision making were assessed through the implementation of both questionnaire (explicit) and computer task (implicit) measures. Questionnaires included Barratt Impulsiveness Scale (BIS-11; Patton et al. 1995) and the Arnett Inventory of Sensation Seeking (AISS; Arnett 1994).The BIS-11 was chosen as it has been validated to have good psychometric properties in both research and clinical environments when quantifying the construct of impulsivity. Likewise, we employed the AISS to measure sensation seeking—an individual tendency closely related to both trait impulsivity and risk-taking (Magid et al. 2007).

Implicit impulsivity was assessed using a Stop-Signal computer task (SST; Logan et al. 1997). Within the SST, participants must respond to an arrow displayed in the centre of the screen either pointing left or right within 500 ms with the ‘b’ and ‘n’ keys, respectively. If the arrow is surrounded with a white circle (go-signal), participants should respond, however, if a red circle (stop-signal) is presented around the arrow the participant must refrain from responding. The SST started with a block of practice trials where participants did not move onto the critical trials until they either completed 20 practice trials without mistake or completed 50 practice trials. Following this, participants completed 50-critical trials. The dependent measures for this task included response time (ms), errors of omission (i.e. failing to respond to a ‘go-signal) and errors of commission (i.e. responding on a ‘stop-signal’). The SST has been shown to have good validity when discriminating between clinical (e.g. attention deficit hyperactivity disorder) and non-clinical (i.e. normative) populations (Lipszyc and Schachar 2010; Solanto et al. 2001).

The Balloon Analogue Risk Task (BART; Lejuez et al. 2002) was used as an assessment of real-world risk taking. In this task, participants are required to inflate an onscreen balloon by pressing the space bar. Each space bar press equates to an increase of £0.05 of virtual currency which can be ‘banked’ by pressing the return key. Each balloon has a randomly allocated tolerance and over inflation will cause the balloon to burst—losing the amount ‘earnt’ in that trial. Due to each balloons threshold being withheld, we could analyse early (pre-experience) responses, as well as learnt responses. The dependent variable is the mean number of pumps on each trial where the balloon did not burst. There was a total of 20 trials in this task.

Decision making was assessed using the Iowa Gambling Task (IGT; Bechara et al. 1994). Here, participants were shown four on screen choices: ‘A’, ‘B’, ‘C’ or ‘D’ and start with a ‘loan’ of £2000 of virtual currency. After each choice, participants are given feedback about their profits and/or losses. Choices ‘A’ and ‘B’ always yield £100, whereas, choices ‘C’ and ‘D’ always yield £50; moreover, for each choice, there is always a 50% chance of having to pay a penalty—the penalty for choices ‘A’ and ‘B’ is always £250, whilst the penalty for choices ‘C’ and ‘D’ is always £50. There was a total of 100 trials in this task, 50 of which are coded to give the participant a ‘fee’. Therefore, this task has two dependent variables: ‘*A-tendency’* (preferring to choose ‘A’ and ‘B’) and *‘B-tendency’* (preferring to choose ‘C’ and ‘D’). Research suggests that more impulsive individuals will have a greater A-tendency due to their tendency to discount the value of delayed rewards (Burdick et al. 2013; Wittmann and Paulus 2008).

### Craving

Both explicit (assessed via questionnaire) and implicit craving (assessed via computer task) levels were measured in this study. Explicit craving was assessed using a 14-item version of the Desires for Alcohol Questionnaire (DAQ; Kramer et al. 2010). Here, a 9-point Likert scale (1 = ‘Disagree completely’: 9 = ‘Agree completely’) was used by participants to rate a series of statements relating to their desire to consume alcohol at the point in time that the questionnaire was administered. The scores attained from the DAQ provide a single measure of craving for each participant, where greater scores specify a greater desire for alcohol consumption. The literature surrounding the psychometric properties of the DAQ has shown that alcoholic patients have a score of ~40, whereas healthy non-alcoholic drinkers score around ~20 (Kramer et al. 2010).

**Fig. 1:**
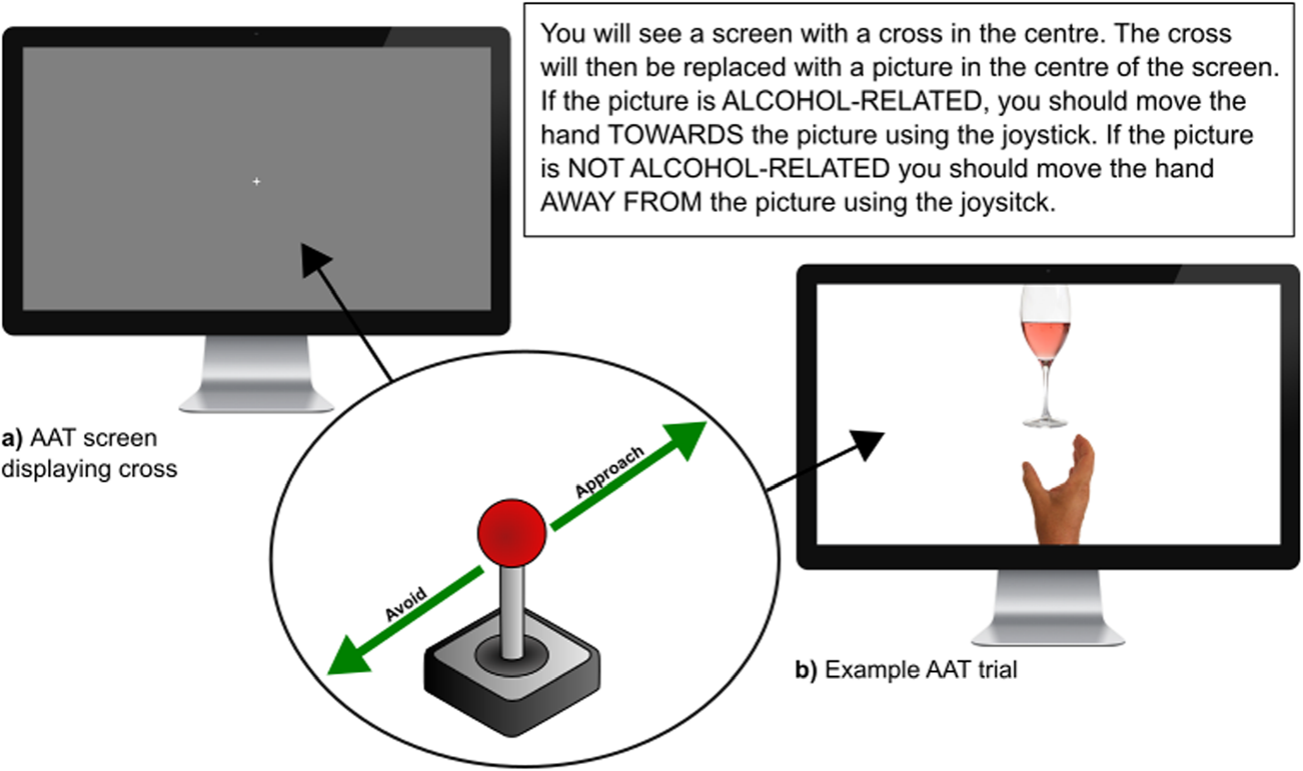
Screenshots of the approach avoidance task administered to participants. AAT Approach Avoidance Task

Implicit craving was assessed using an approach-avoidance task (AAT; Rinck and Becker 2007; Wiers et al. 2010; see Fig. 1). Previous versions of this task have used a different model, where ‘pulls’ on a joystick result in the image being moved closer to the participant, and ‘pushes’ making the image move away. In the current version, we required participants to move an image of a hand located at the bottom of the screen either towards (approach) or away from (avoid) an image positioned at the top of the screen using a joystick. On each trial a fixation cross was presented in the centre of the screen for 1 s. There were two conditions ‘approach alcohol’ and ‘avoid alcohol’, with a total of 64 trials in each, of which 32 were critical trials (pictures related to alcohol, e.g. a pint of beer) and 32 trials were control trials (pictures not-related to alcohol, e.g. pint of water). In the ‘approach alcohol’ condition, participants were required to approach alcohol-related images by moving the joystick towards the screen and vice versa in the ‘avoid alcohol’ condition. The order that participants completed conditions was counterbalanced. The dependent variables are response time (ms) and number of errors made. It is hypothesised that participants with a greater craving for alcohol have a lower response time when approaching alcohol-related pictures vs non-alcohol-related pictures and a slower response time when avoiding alcohol-related pictures vs non-alcohol-related pictures. As this version of the AAT was, as yet, unvalidated as a measure of implicit craving, this study represented an initial validation of the method.

### Voluntary drinking

Incentive motivation for alcohol was assessed using a progressive ratio schedule task (PRS; Vezina 2004; Ward et al. 2006). Participants pressed the space bar on a computer keyboard to ‘earn’ a up to a total of 12 5-ml shots of 37% ABV vodka diluted in 20 ml of mixer (e.g. coke, lemonade, tonic). For each subsequent drink, participants’ response requirement doubled; i.e. the first shot was delivered following one spacebar press; the second shot, following two spacebar presses; and the third following four presses. After each drink, participants rated pleasantness on a 15-point Likert scale (1 = ‘Very unpleasant’: 15 = ‘Very pleasant’). The dependent variable in this task was the number of drinks earned by each participant and their subjective enjoyment of consuming each drink earnt.

### Physiological parameters

Heart rate (HR) and inter-beat interval (IBI) data were collected throughout the procedure using a Polar A300 Activity Tracker and a Polar H7 Heart Rate Sensor (Polar Electro, Finland). Research suggests that unbound sC is highly correlated with serum cortisol (Daniel et al. 2006; Dorn et al. 2007; Eatough et al. 2009), thus provides a reliable measure of hypothalamic pituitary adrenocortical axis (HPA) activation. In addition, research suggests that sAA release has a strong relationship with noradrenaline release (Chatterton et al. 1996; Thoma et al. 2012), which allows for a non-invasive bio-marker of sympathetic adrenomedullary axis (SAM) activation. Saliva samples (2 ml of passive drool) were taken twice. In the stress condition, they were taken before and after the Trier Social Stress Test (TSST; Kirschbaum et al. 2008). In the control condition, saliva samples were taken before and after sitting quietly for 15 min in the lab. Samples were placed on ice, then centrifuged (3000 × 15 min) and the supernatant was split into two 1-ml aliquots and frozen (− 20 °C) until assay. Samples were analysed in the laboratory using Salimetrics sC and sAA enzyme-linked immunosorbent assay (ELSIA) kits (Stratech, Ely, UK).

### Procedures

Questionnaires and the SST were programmed using PsyToolKit (Stoet 2010, 2017), and the BART, AAT and PRS were programmed and executed using PsychoPy software (Peirce 2007, 2008).

#### Phase 1: baseline assessments

Once participants suitability was confirmed via the pre-screening questionnaire, participants were randomly placed into either the experimental or control group and invited to attend a laboratory session lasting approximately 90 min. All study sessions took place between 11:00 and 15:30 to minimise the effects of the diurnal slopes of cortisol levels on our observations (Stone et al. 2001). Upon arrival, participants were given the opportunity to re-read the previously e-mailed information sheet and ask any questions. Two identical consent forms were then signed by the participants, one they could keep and the other was kept by the principal experimenter in a secure master file. The Polar A300 Activity Tracker and H7 Heart Rate Sensor was then fitted and recording was started. Participants then completed the unit/week questionnaire, AUDIT, BDS, BIS-11, AISS and (pre-intervention) DAQ on the computer. Subsequently, participants completed three computer tasks: SST, BART and (pre-intervention) AAT and provided the pre-intervention saliva sample. The order by which questionnaires and computer tasks were administered were counterbalanced to eliminate order effects.

#### Phase 2: the stress challenge

##### Stress group (TSST)

The procedure for participants in the stress group was made up of two stages: (A) preparation/anticipation and (B) the stress challenge. A shortened version of the TSST was used because participant feedback and HR data obtained through preliminary studies suggested that participants found the math portion of the TSST to not be as stressful as the speech. At the beginning of stage A, participants were told that they would have 10 min to prepare a 5-min speech about their dream job and what made them an ideal candidate. During this time, participants could plan their speech and makes notes on a piece of paper; however, they were informed that the speech must be performed without notes. During stage B, participants were led to a room containing a panel of three strangers wearing lab coats, sat behind a table and a video camera. The participant was then asked to deliver their speech to the panel. During this time, one member of the panel made notes using a clipboard. If the participant ceased talking for more than 20 s, they were asked to continue and reminded of how much time remained of their 5-min slot. At the end of stage B, following the 15-min stress-test procedure, a post-intervention saliva sample was taken.

##### Control group

Participants in the control group did not complete a stress challenge. Therefore, during stages A and B, participants had a 15-min break in the waiting room. After the 15-min break, participants provided their post-intervention saliva sample.

#### Phase 3: post-stress

Participants completed a post-intervention DAQ and AAT. They were then invited to take part in the PRS. Any participant who could not take part or did not wish to take part moved directly to debriefing.

Participants were fully debriefed and informed that the video recordings of their speech were to be destroyed and that their saliva samples would be rendered acellular within a 24-h period in accordance with the Human Tissues Act. Any participant that had consumed alcohol was advised to remain in the waiting area for 15 min to allow time for any intoxicating effects to wear off. Finally, participants had another opportunity to ask any questions.

### Data preparation and statistical analysis

All data were analysed using R (version 3.4.4) and IBM SPSS (version 24) for Windows. IBI data acquired from the Polar Heart Rate monitor were converted standard deviation of NN intervals (SDNN) using Kubios (version 3.1) for Windows (Tarvainen et al. 2014). For all parametric analyses, studentised residuals were examined and outliers (> 1.5*IQR) removed prior to analysis (< 1% observations). All variables were initially compared for sex differences using a series of independent sample *t* tests. ‘Reactivity’ (reactivity = speech − pre) and ‘Recovery’ (recovery = speech − post) variables were calculated for HR and SDNN data. ‘Change’ (change = post − pre) variables were calculated for DAQ, AAT score (where AAT score = median avoid reaction time – median approach reaction time), sC and sAA. A single score for IGT was also calculated (IGT score = a tendency − b tendency, where higher scores = more advantageous decision-making). For repeated measures that were relevant to stress (pre- and post-TSST), we refer to the within-subject effects as ‘time’; this refers to pre- and post-TSST for the stress group and pre-vs post-non-stress (sitting quietly in the waiting room) for the control group.

As a manipulation check, physiological responses to the TSST were examined using 2 × 2 mixed-design analyses of variance (ANOVA; two-level within-subject factor = time [pre, post]; two-level between-subject factor = group [stress, control]). The effects of the TSST on change in craving (DV) were examined using independent *t* tests on both AAT change (post-pre) and DAQ change (post-pre) using group (stress, no stress) as the IV in both tests. In order to examine variables that predict alcohol consumption in the PRS task, we grouped covariates into several categories: (1) physiological responses to stress (HR, SDNN, sC, sAA, group); (2) craving (DAQ change, AAT change); (3) risk-taking, sensation seeking (BART, AISS, IGT) and impulsivity (BIS and SST); (4) prior alcohol use (AUDIT, units/week, BDS); and (5) drink enjoyment. To determine the effects of stress on the number of drinks consumed in the PRS task, we fitted these groups of covariates to several negative binomial regression models (PRS data were overdispersed count data). In addition to the covariates, ‘group’ (stress vs no stress) and gender were added to all models as fixed factors. To simplify regression models we applied backwards elimination, sequentially removing non-significant terms with the largest *p* values (> 0.05) until only significant effects remained (< 0.05). All significant covariates from the subcategories were then entered into a final negative binomial regression model to determine the best model for predicting alcohol consumption. Descriptive statistics are reported as mean ± SEM.

## Results

### Sample characteristics

Table 1 displays the sample characteristics, in terms of prior alcohol use behaviour, personality traits, craving (pre-stress and post-stress) and drinking. Sex differences were found for two variables. BIS Motor was greater in males (M = 24.32, SD = 3.85) than in females (M = 21.88, SD = 2.67), *t* (37) = 2.33, *p* = 0.02, Cohen’s *d* = 0.72 [95% CI of difference 0.3–4.6]. AISS novelty was also greater in males (M = 28.86, SD = 2.8) than in females (M = 26.18, SD = 3.26), *t* (32) = 2.71, *p* = 0.01, Cohen’s *d* = 0.89 [95% CI of difference 0.7–4.7].

**Table 1:** Demographic characteristics of sample, and raw data

	Male (SD)	Female (SD)	Total (SD)
Demographic			
Age	23.45 (4.78)	24.53 (5.14)	23.92 (4.90)
*N*	22	17	39
Alcohol use			
AUDIT	9.86 (5.38)	8.20 (6.06)	9.17 (5.65)
BDS	7.45 (3.95)	5.53 (3.56)	6.62 (3.86)
Units/week	20.41 (14.26)	14.76 (20.64)	17.95 (17.31)
Personality traits			
BART	24.88 (12.89)	25.55 (15.68)	25.17 (13.98)
BIS Attentional	17.36 (4.56)	16.59 (3.26)	17.03 (4.02)
BIS Motor	24.32 (3.85)*	21.88 (2.67)*	**23.26 (3.56)**
BIS Nonplanning	23.59 (5.04)	22.71 (4.96)	23.21 (4.96)
AISS Novelty	28.86 (2.8)*	26.18 (3.26)*	**27.69 (3.26)**
AISS Intensity	26.77 (3.26)	24.00 (4.97)	27.69 (3.26)
IGT Score	7.73 (43.72)	11.00 (30.08)	0.44 (39.06)
SST Mean RT	367.77 (121.94)	351.47 (42.1)	360.66 (95.03)
SST Error of Commission	3.55 (4.65)	2.35 (2.23)	3.03 (3.79)
SST Error of Omission	34.18 (15.15)	40.53 (16.62)	36.95 (15.92)
Physiology			
HR pre	79.35 (12.39)	79.87 (15.06)	79.60 (13.51)
HR anticipation	80.44 (11.63)	82.43 (16.60)	81.35 (13.96)
HR speech	90 (17.20)	91.25 (21.39)	90.59 (19.02)
HR post	78.38 (10.30)	78.31 (13.36)	78.34 (11.63)
SDNN pre	21.18 (9.95)	18.49 (3.95)	19.91 (7.74)
SDNN anticipation	22.31 (7.28)	19.18 (5.4)	20.87 (6.59)
SDNN speech	20.12 (7.64)	21.09 (6.67)	20.58 (7.11)
SDNN post	20.80 (5.81)	20.32 (6.64)	20.58 (6.12)
Cortisol pre	0.20 (0.15)	0.25 (0.16)	0.22 (0.15)
Cortisol Post	0.17 (0.01)	0.22 (0.16)	0.19 (0.09)
Alpha-amylase pre	39.53 (45.95)	52.02 (53.10)	45.27 (49.06)
Alpha-amylase post	55.93 (86.36)	90.15 (71.75)	71.65 (47.94)
Craving			
DAQ pre	38.27 (13.96)	32.76 (20.63)	35.87 (17.16)
DAQ post	40.50 (18.26)	39.12 (25.95)	39.89 (21.68)
AAT pre	0.02 (0.07)	0.02 (0.09)	0.02 (0.7)
AAT post	0.03 (0.05)	0.03 (0.10)	0.03 (0.08)
Drinking			
Number of drinks consumed	2.45 (3.53)	3.41 (3.76)	2.87 (3.61)
Drink enjoyment	9.57 (2.63)	8.54 (2.25)	9.09 (2.46)

*AUDIT* Alcohol Use Disorders Identification Test, *BDS* Binge Drinking Scale, *BART* Balloon Analogue Risk Task, *BIS* Barratt Impulsiveness Scale, *AISS* Arnett Inventory of Sensation Seeking, *IGT* Iowa Gambling Task, *SST* Stop Signal Task, *HR* heart rate, *SDNN* standard deviation of NN intervals, *DAQ* Desires for Alcohol Questionnaire, *AAT* Approach Avoidance Task

All significant effects are highlighted in bold

*p  0.05

### Bivariate analysis

To assess the reliability of our measures and to attempt to validate the AAT as an assessment of implicit craving, we examined intercorrelations of measures of prior alcohol use, personality traits, craving and consumption, and stress reactivity were tested by completing Pearson correlations between each subset of variables previously listed. All measures of prior alcohol use behaviour were significantly intercorrelated (all *r*_*s*_ = 0.27–0.81, all *p*_s_ < 0.05; data not shown). As shown in Table 2, several personality traits were intercorrelated. Motor impulsivity (BIS) was positively correlated with sensation seeking (AISS novelty and intensity), and negatively correlated with omission errors on the SST. However, motor impulsivity was not significantly correlated with either decision making (IGT) or risk-taking (BART). There was a trend for a positive correlation between SST and BART which approached significance (*p* = 0.055), as well as a trend for a negative correlation between IGT and non-planning BIS (*p* = 0.087). Although both explicit (DAQ) and implicit (AAT) craving pre- and post-scores were correlated, the two measures were not intercorrelated (Table 3), suggesting low reliability. A number of physiological and stress-reactivity parameters were intercorrelated (Table 4): HR reactivity was positively correlated with HR recovery; HR recovery was negatively correlated with SDNN recovery. There was a negative correlation between SDNN recovery and HR recovery that approached significance (*p* = 0.06). SDNN reactivity was positively correlated with SDNN recovery, and there was a trend towards a negative correlation with sAA change (*p* = 0.06).

**Table 2:** Inter-correlations (Pearson’s *R* values) of personality trait assessments

	1	2	3	4	5	6	7	8
1. BIS Attentional	–							
2. BIS Motor	0.42**	–						
3. BIS Non-Planning	0.53**	0.42**	–					
4. AISS Novelty	0.09	0.34*	0.25	–				
5. AISS Intensity	0.37*	0.33*	0.23	0.38*	–			
6. IGT Score	0.14	0.05	− 0.28^†^	− 0.10	0.10	–		
7. SST Omission Error	− 0.22	− 0.36*	− 0.21	− 0.22	− 0.12	0.03	–	
8. SST Commission	− 0.15	− 0.02	− 0.19	0.15	− 0.07	− 0.16	− 0.51**	–
9. BART	0.20	− 0.03	0.22	0.10**	0.44	− 0.14	− 0.21	0.31^†^

*BIS* Barratt Impulsiveness Scale, *AISS* Arnett Inventory of Sensation Seeking, *IGT* Iowa Gambling Task, *SST* Stop Signal Task, *BART* Balloon Analogue Risk Task

*p < 0.05, **p < 0.01, ^†^*p* < 0.09

**Table 3:** Inter-correlations (Pearson’s *R* values) of craving measures

	1	2	3	4	5
1. DAQ Pre-TSST	–				
2. DAQ Post-TSST	0.89**	–			
3. DAQ change (post − pre)	0.18	0.61**	–		
4. AAT Pre-TSST	− 0.01	0.08	0.22	–	
5. AAT Post-TSST	0.00	0.12	0.24	0.47**	–
6. AAT change (post − pre)	0.01	0.04	0.03	− 0.50**	0.54**

*DAQ* Desires for Alcohol Questionnaire, *AAT* Approach Avoidance Task

*p < 0.05, **p < 0.01

**Table 4:** Inter-correlations (Pearson’s *R* values) of physiological biomarkers of stress

	1	2	3	4	5
1. HR reactivity	–				
2. HR recovery	0.88**	–			
3. SDNN reactivity	− 0.18	− 0.32^†^	–		
4. SDNN recovery	− 0.30	− 0.39*	0.49**	–	
5. Alpha-amylase change	− 0.19	− 0.07	− 0.32^†^	− 0.01	–
6. Cortisol change	0.14	0.11	0.07	0.04	0.19

*HR* heart rate, *SDNN* standard deviation of NN intervals

*p < 0.05, **p < 0.01, ^†^*p* < 0.09

### Manipulation check

**Fig. 2:**
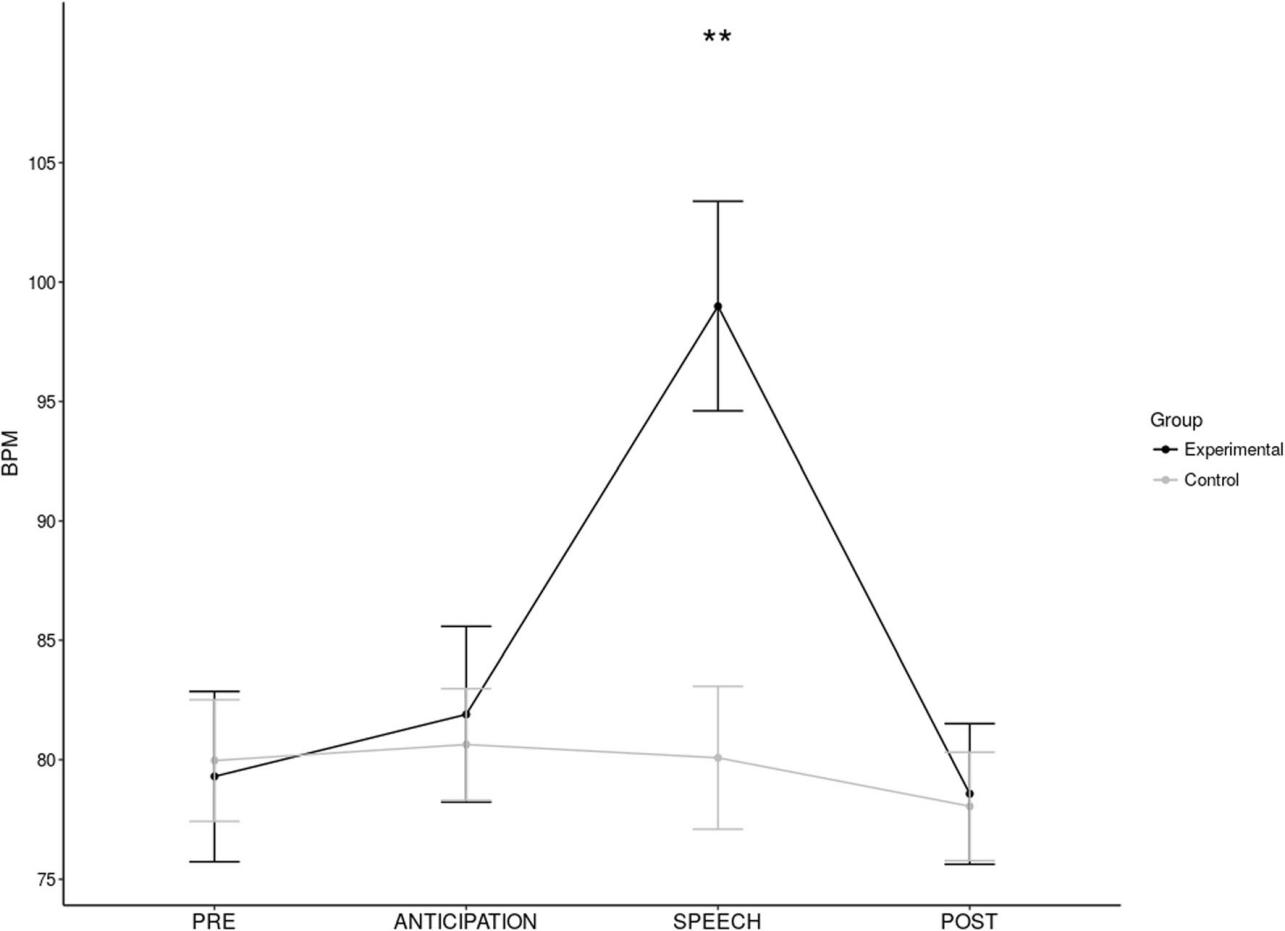
Mean (± SEM) change (post-intervention − pre-intervention) Desires for Alcohol Questionnaire (DAQ) scores (higher score = higher desire) in ‘stress’ and ‘no-stress’ groups. *p ≤ 0.05

Responses to the TSST were assed using various physiological measures including HR, heart rate variability (SDNN), sC and sAA. A two-way repeated measures ANOVA was carried out on HR data. According to the Mauchley’s test, the assumptions of sphericity have been violated, so we adopted Greenhouse-Geisser-adjusted degrees of freedom. There was a significant main effect of time, *F* (1.96, 62.78) = 29.61, *p* < 0.001, η_p_^2^ = 0.48, but no significant main effect of group, *F* (1,32) = 1.66, *p* = 0.2. There was a significant time x group interaction, *F* (1.96, 62.78) = 25.03, *p* < 0.001, η_p_^2^ = 0.44 (Fig. 2), characterised (Bonferroni-corrected pair-wise comparisons) as the experimental group showing significantly higher HR at the time of the speech when compared to baseline (*p <* 0.001), anticipation (*p* < 0.001) and post-stress readings (*p* < 0.001). There were no changes in HR for the control group (all *p*_s_ > 0.1). There was no main effect on SDNN of time (*F* < 1) or of group, *F* (1,32) = 2.18, *p* = 0.15, nor was there a significant time × group interaction (*F* < 1). The main effect of time on sC approached significance, *F* (1, 34) = 3.826, *p* = 0.059. There were no effects of group (*F* < 1) or a time × group interaction, *F* (1, 34) = 1.99, *p* = 0.168. The main effect of time on sAA approached significance, *F* (1, 35) = 3.33, *p* = 0.077. There was no main effect of group, *F* (1,35) = 2.24, *p* = 0.14, and no time × group interaction (*F* < 1).

### Effects of psychosocial stress on craving

**Fig. 3:**
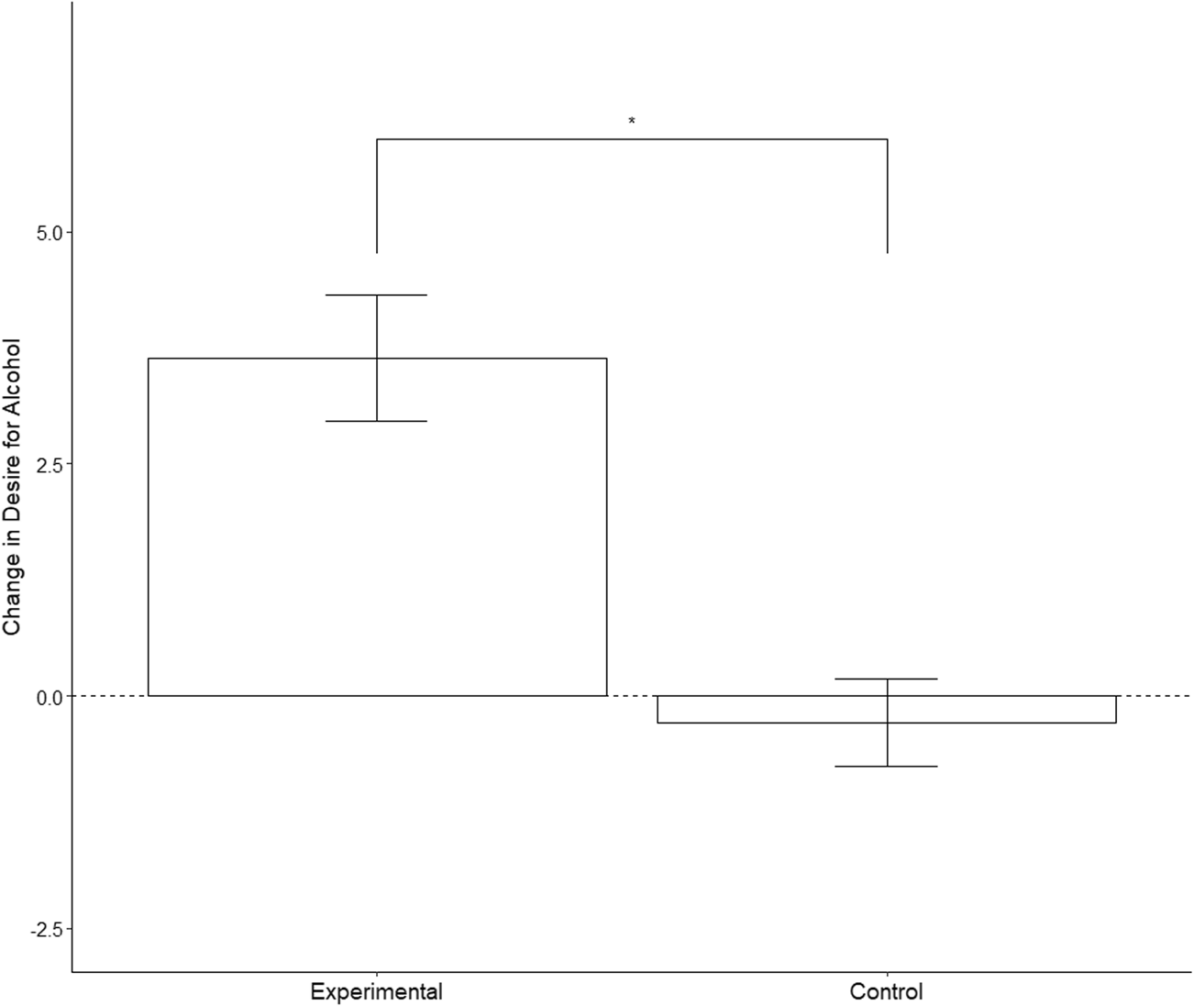
Mean (± SEM) change in physiological arousal (heart rate) prior to, during and following the intervention in both ‘stress’ and ‘no-stress’ groups. BPM beats per minute. **p ≤ 0.01

To test the hypothesis that an acute psychosocial stressor would increase craving, we examined change scores (change = post − pre) for both explicit (DAQ change) and implicit (AAT change) using two independent *t* tests with group as the independent variable. Explicit craving (DAQ) change was significantly greater (Fig. 3) in the stress group when compared to the control group, *t*(33) = 2.44, *p* = 0.02, Cohen’s *d* = 0.85 [95% CI of difference = 1.26–13.95]. There was no significant group difference between implicit craving (AAT) change, *t* (36) = − 0.93, *p =* 0.36.

### Effects of psychosocial stress on drinking

Negative binomial linear regression models were fitted to determine which covariates best predicted number of alcoholic drinks consumed. Table 5 displays the results of the final regression model following backwards elimination. The steps of the backwards elimination are found in the supplementary data.

**Table 5:** Summary of final negative binomial regression model predicting alcohol consumption following backwards elimination

Variable	β S.E.	β	*z*
Constant	1.29	0.54	2.41**
Group (control)	− 0.80	0.27	− 2.93**
IGT score (a tendency − b tendency)	− 0.01	0.003	− 3.08**
HR recovery	− 0.04	0.01	− 3.26**
SDNN reactivity	0.04	0.02	2.75**
SDNN recovery	− 0.05	0.03	− 2.15*
Mean drink enjoyment	0.09	0.05	2.01*

AIC = 101.98

*IGT* Iowa Gambling Task, *HR* heart rate, *SDNN* standard deviation of NN intervals

*p < 0.05, **p < 0.01

## Discussion

The primary aim of this study was to examine the effects of psychosocial stress on craving and voluntary alcohol consumption in healthy social drinkers. The study was developed with incentive sensitisation and neurocognitive endophenotypes as the theoretical framework and aimed to test predictions from both theories. Specifically, we tested the hypothesis that neurocognitive endophenotypes, such as impulsivity and risk-taking, affect stress-induced alcohol consumption in healthy social drinkers. First, we found that a mild psychosocial stressor increased voluntary alcohol consumption in healthy social drinkers. We also found that, although stress increased both craving and drinking, these two measures were statistically independent, with those that showed higher craving not necessarily consuming more alcohol. Instead, we found that risky decision making (IGT) and physiological stress-reactivity (SDNN and HR) were the most important predictors of alcohol consumption following psychosocial stress, and that those who drank more reported higher levels of subjective enjoyment of the drink.

Previous research has provided evidence that stress-reactivity plays a crucial role in the onset of relapse within abstinent alcoholics (Sinha et al. 2011; Sinha 2012), as well as an increase in craving (alcohol-seeking) and self-administration of alcohol in translational (animal) studies (Cleck and Blendy 2008; Koob and Kreek 2007; Koob et al. 2004; Rasmussen et al. 2006). Our data support and extend these findings, showing that participants with greater HRV reactivity and poor HR and HRV recovery following stress tended to drink more. This is, to the best of our knowledge, the first empirical demonstration of how individual differences in stress reactivity and recovery affect voluntary drinking. This finding is supported by the increasing body of work focusing on vagal dysfunction, and its inhibitory effect on the management of allostatic load (Ingjaldsson et al. 2003; Rechlin et al. 1996; Shively et al. 2007; Thayer and Sternberg 2006), suggesting that those with poor vagal tone may be at increased risk of stress-induced craving for alcohol (Koob and Le Moal 2001). Further assessments of stress-reactivity (i.e. sC and sAA) did not reveal any significant effects of the TSST. Other studies have consistently found the TSST to increase neuroendocrinal responses to stress (Het et al. 2009; Kelly et al. 2008; Kirschbaum et al. 1993; Oswald et al. 2006). The blunted sC and sAA responses observed in our data could have been the result of the omission of the mental arithmetic test from our TSST procedure (on the basis of participant feedback acquired during preliminary studies). The reduction of the TSST running time from 10 to 5 min may not have given adequate time for cortisol and alpha-amylase to be detected through saliva. Regardless of this, the shorted TSST used in this study still provoked a significant increase in HR (both during anticipation and during the speech) and increased both craving and alcohol consumption.

We found that risky decision-making (in the IGT) was a significant predictor of alcohol consumption. Risky or impulsive decision-making has been previously associated with AUD, with abstinent alcoholics in particular showing deficits in their decision making ability (Fein et al. 2004). Risky-decision making is also observed in those who misuse other substances (Barry and Petry 2008), including cocaine (Bolla et al. 2003; Verdejo-Garcia et al. 2007) and cannabis (Hermann et al. 2009; Verdejo-Garcia et al. 2007). Furthermore, our previous research has shown that risk-taking was positively correlated with alcohol craving (Clay et al. 2018) and others have demonstrated links to reported alcohol use in social drinkers (Fernie et al. 2010). We did not find that any other measures of inhibitory control (stop-signal task, BIS) were predictive of voluntary alcohol consumption. McGrath, Jones and Field (2016) examined the effects of a psychosocial stressor on voluntary drinking in a semi-naturalistic ‘mock bar’ environment. They hypothesised that alcohol consumption in this setting would be increased following stress owing to changes in inhibitory control. Similar to our study, they found that stress increased drinking. Also similar to our findings, they did not find any evidence that impulse control (via the stop-signal task) was predictive of drinking. However, here we observed that one measure of inhibitory control, risky-decision making, was predictive of drinking. Collectively, these data suggest that stress-induced lapses in inhibitory control are not sufficient to explain variability in drinking, but instead lend support to the theory that subtle neurocognitive endophenotypes may be more important in predicting the move from controlled to uncontrolled drinking.

Our data provides evidence that explicit craving was increased following a psychosocial stressor in a sample of healthy social drinkers, replicating our prior work (Clay et al. 2018). Interestingly, alcohol craving was not found to be a significant predictor of alcohol consumption, although explicit craving (DAQ) change was greater in the stress group. The literature suggests that craving is the prominent risk factor in the onset of relapse, and there is a large surrounding research output in support of this idea (e.g. Litt et al. 2000; Robinson and Berridge 1993; Schneekloth et al. 2012; Sinha et al. 2011). The fact our data did not substantiate this could be due to craving only being an important predictor of alcohol consumption in those already addicted, i.e. alcoholics. Consequently, our data suggests that decision-making is of a higher importance when predicting alcohol consumption in healthy social drinkers. If this is the case, underlying personality traits could be key in predicting those most at risk of moving from a social (habitual) state to a dangerous (addicted) one. It should be considered that had we used different cues that grabbed the attention or a different assessment of implicit craving, there may have been a different outcome in terms of implicit craving change within social drinkers. Clinical research indicates that both addicted cocaine and alcohol users tend to show an increased craving following presentation of stress-inducing imagery (Sinha et al. 2011). Incentive sensitisation theory (e.g. Robinson and Berridge 1993) predicts that the physiological response to stress acts as a cue that induces incentive motivation to take the drug; in other words, incentive sensitisation predicts that stress will increase craving. However, incentive sensitisation theory suggests that craving will also be increased by a number of environmental cues (drug stimuli, for example), and this has been demonstrated in many studies (e.g. Field and Powell 2007; Field and Quigley 2009; Wiers et al. 2006). For future studies, it would be useful to include a number of additional cues (e.g. drug-related visual stimuli, or olfactory/gustatory cues) to examine their role of impulsivity/risk-taking on craving/consumption of alcohol. It may be that the stressor used here was insufficient to induce incentive motivation to consume alcohol, thus explaining why craving did not predict drinking. Finally, although we found differences in explicit craving, we did not find differences in our measure of implicit craving. It may be that the AAT was not sensitive enough to detect changes in craving in our healthy sample. Others (e.g. Field and Powell 2007) have used attentional bias protocols as a measure of implicit craving, and this may be more sensitive. The AAT was also not intercorrelated with our assessment of explicit craving (DAQ), suggesting that the AAT may not have good psychometric properties for the assessment of implicit craving of alcohol. In future studies, other measures of implicit craving, such as the attentional bias measures (Field and Powell 2007), could be included in addition to the AAT. However, the attentional bias task take a long time to carry out, and in the current context (i.e. following stress), this is not ideal. Future research should strive to validate a novel, less temporally limited, assessment of implicit craving.

An additional note from our data was that ‘drink enjoyment’ was not significantly correlated with craving. This finding is consistent with the predictions of incentive sensitisation theory (Robinson and Berridge 1993), which states that that incentive motivation to drink (wanting) and subjective enjoyment (liking) are independent. However, interestingly, we observed that there was a strong correlation between subjective ‘liking’ of alcohol and voluntary alcohol consumption. This finding further suggests that, in healthy social drinkers that are primed with an acute risk-factor for AUD (psychosocial stress), the predictions of incentive sensitisation theory do not stand. Instead, those at highest risk of stress-induced alcohol consumption did appear to be those that are more stress reactive, have poorer vagal tone (lower stress-recovery) and are higher in risky decision making. This may have significant implications in the search for sub-clinical early identification of those at risk of AUD.

There were several limitations to the present study that should be considered. First, the generalisability of the findings is limited by our sample, who were students and staff at the University of Portsmouth and may not have been truly representative of the general population. Second, we used a modified version of the TSST, which did not induce significant changes in either salivary cortisol or saliva alpha amylase, as shown by numerous previous studies. However, we did observe significant increases in HR (and in HRV parameters) following the modified version of the test, suggesting that it did induce stress in the participants. It may be that a longer version of the TSST would have increased craving and this may have been linked to drinking. A further limitation of this study was the fact that both stress and control groups completed cognitive trials. Dickerson and Kemeny (2004) suggest that cognitive tasks stimulate the HPA axis enough to detect changes cortisol levels. Therefore, the use of cognitive tasks as a means of quantification within stress research could act as a confounding variable. Ideally, research in this area should aim to assess stress variability and individual differences on separate occasions. Unfortunately, due the nature of this study, mitigating against such a confound in this way was not an option due to the need to measure craving/consumption following stress. In the same vein, the IGT has received some criticism of its validity (Dunn et al. 2006; Steingroevera et al. 2013). Bechara et al. (1994) make several assumptions about the decision-making processes in healthy participants: ‘Healthy participants learn to prefer the good options over the bad options, healthy participants show homogeneous choice behaviour and healthy participants first explore the different options and then exploit the most profitable ones’. A recent study (Steingroevera et al. 2013) suggests instead that ‘Healthy participants often prefer decks with infrequent losses, healthy participants show idiosyncratic choice behaviour and healthy participants do not show a systematic decrease in the number of switches across trials’. These findings suggest that decision-making, when assessed using the IGT, in healthy participants is heterogeneous. Therefore, future research using should err on the side of caution when using the IGT. Nevertheless, our data suggests that this heterogeneity can predict variability in alcohol consumption amongst social drinkers. Additionally, both groups of participants drank less than half of the 12 available drinks, meaning that on average < 1 unit of alcohol was consumed, which may indicate an unsuccessful stress manipulation following the modified TSST. However, as previously stated, we believe that the stress manipulation was successful due to the significant HR group × time interaction and that the insignificant sC and sAA responses were due to shortening of the TSST. Furthermore, our method of assessing drink enjoyment, i.e. via 15-point Likert scale, may have limited subject interest. Finally, the alcohol used in the voluntary drinking procedure was vodka mixed with a mixer of choice. It may be that if we had given a greater range of drinks as reinforcers, this would have increased the amount consumed in some participants. We chose to use vodka and mixer owing to the very precise amount (and *v*/*v* of alcohol) this afforded us. In future studies, we may include a choice of beer, wine and spirit to examine if there are any differences observed. However, subjectively, no participants voiced this as a reason for not consuming the alcohol, so we do not think this represented significant confound.

In conclusion, we have demonstrated that psychosocial stress causes an increase in voluntary alcohol consumption. We also demonstrated, for the first time, that the number of drinks consumed was predicted by greater physiological reactivity and slower physiological recovery following stress, and by risky decision making. Collectively, our findings may provide a useful translational framework through which we can further study the early risk factors that predict the shift from controlled recreational drinking and alcohol misuse, leading (in some) to AUD. In particular, the role of variation in personality traits and physiological reactivity that predict cue-induced alcohol consumption needs to be further studied in order that we can be in a stronger position to identify those at risk of developing AUD in the future. A useful target of study in the first instance may be role of variation in efficacy of neural circuits that underlie both stress-reactivity and impulse control (i.e. cortico-amygdala-striatal projections; Koob 2008) in cue-induced drinking.

## Electronic supplementary material

ESM 1 (DOCX 29.6 kb)
