# Supplementary material for: The role of stress-reactivity, stress-recovery and risky decision-making in psychosocial stress-induced alcohol consumption in social drinkers
Source: Psychopharmacology (Berl). 2018 Sep 12;235(11):3243–57. doi: 10.1007/s00213-018-5027-0 (PMC6208948; doi:10.1007/s00213-018-5027-0)
Supplement: Supplementary file 1 — (DOCX 29.6 kb) [file 213_2018_5027_MOESM1_ESM.docx]

**Table 6.** Summary of negative binomial regression models of variables related to physiological responses to stress.

| Variable |  | β | S.E. β | z |
| --- | --- | --- | --- | --- |
| *Step 1* |  |  |  |  |
| Constant |  | 2.45 | 0.52 | 4.73 |
| HR Reactivity✝ |  | 0.05 | 0.03 | 1.66 |
| HR Recovery** |  | -0.11 | 0.04 | -2.95 |
| Cortisol Change |  | -0.44 | 1.7 | -0.26 |
| Amylase Change |  | -0.002 | 0.002 | -0.99 |
| SDNN Reactivity |  | 0.04 | 0.03 | 1.18 |
| SDNN Recovery** |  | -0.12 | 0.04 | -2.98 |
| Group (Control)** |  | -2.00 | 0.54 | -3.74 |
| Gender (Female) |  | 0.22 | 0.36 | 0.60 |
| *Step 2* |  |  |  |  |
| Constant |  | 2.41 | 0.55 | 4.42 |
| HR Reactivity✝ |  | 0.05 | 0.03 | 1.67 |
| HR Recovery** |  | -0.11 | 0.04 | -2.83 |
| Amylase Change |  | -0.002 | -0.002 | -0.82 |
| SDNN Reactivity* |  | 0.06 | 0.03 | 2.23 |
| SDNN Recovery** |  | -0.14 | 0.04 | -3.35 |
| Group (Control)** |  | -1.76 | 0.53 | -3.29 |
| Gender (Female) |  | 0.45 | 0.36 | 0.13 |
| *Step 3* |  |  |  |  |
| Constant |  | 2.42 | 0.54 | 4.53 |
| HR Reactivity✝ |  | 0.05 | 0.03 | 1.69 |
| HR Recovery** |  | -0.11 | 0.04 | -2.86 |
| Amylase Change |  | -0.001 | 0.002 | -0.80 |
| SDNN Reactivity* |  | 0.06 | 0.03 | 2.28 |
| SDNN Recovery** |  | -0.14 | 0.04 | -3.32 |
| Group (Control)** |  | -1.76 | 0.53 | -3.38 |
| *Step 4* |  |  |  |  |
| Constant |  | 2.26 | 0.51 | 4.47 |
| HR Reactivity✝ |  | 0.06 | 0.03 | 1.91 |
| HR Recovery** |  | -0.11 | 0.04 | -2.88 |
| SDNN Reactivity* |  | 0.07 | 0.03 | 2.40 |
| SDNN Recovery** |  | -0.13 | 0.04 | -3.31 |
| Group (Control)** |  | -1.64 | 0.53 | -3.08 |

HR, heart rate; SDNN, standard deviation of NN intervals. * *p* <.01, ** *p* < .05, ✝ *p* <.09.

**Table 7.** Summary of negative binomial regression models of variables related to craving

| Variable |  | β | S.E. β | z |
| --- | --- | --- | --- | --- |
| *Step 1* |  |  |  |  |
| Constant |  | 1.09 | 0.37 | 2.96 |
| DAQ Change |  | 0.03 | 0.03 | 1.07 |
| AAT Change |  | -1.88 | 3.00 | -0.63 |
| Gender (Female) |  | 0.04 | 0.05 | 0.07 |
| Group (Control) |  | -0.32 | 0.54 | -0.58 |
| *Step 2* |  |  |  |  |
| Constant |  | 1.11 | 0.33 | 3.37 |
| DAQ Change |  | 0.03 | 0.03 | 1.18 |
| AAT Change |  | -1.91 | 3.00 | -0.64 |
| Group (Control) |  | -0.32 | 0.53 | -0.60 |
| *Step 3* |  |  |  |  |
| Constant |  | 0.99 | 0.25 | 3.95 |
| DAQ Change |  | 0.04 | 0.03 | 1.52 |
| AAT Change |  | -2.56 | 2.98 | -0.86 |
| *Step 4* |  |  |  |  |
| Constant |  | 0.98 | 2.52 | 3.90 |
| DAQ Change |  | 0.04 | 0.03 | 1.54 |

DAQ, Desires for Alcohol Questionnaire; AAT, Approach Avoidance Task.

**Table 8.** Summary of negative binomial regression models of variables related to risk-taking, sensation seeking (BART, AISS, IGT) and impulsivity (BIS and SST).

| Variable |  | β | S.E. β | z |
| --- | --- | --- | --- | --- |
| *Step 1* |  |  |  |  |
| Constant |  | 0.42 | 3.09 | 0.14 |
| BART |  | 0.004 | 0.02 | 0.24 |
| AISS Intensity |  | -0.01 | 0.07 | -0.20 |
| AISS Novelty |  | -0.04 | 0.09 | -0.45 |
| IGT Score |  | -0.01 | 0.01 | -1.24 |
| BIS Attentional |  | 0.09 | 0.07 | 1.27 |
| BIS Motor |  | -0.05 | 0.08 | -0.68 |
| BIS Non-planning |  | 0.04 | 0.07 | 0.60 |
| SST Commission |  | 0.08 | 0.08 | 0.91 |
| SST Omission |  | 0.01 | 0.02 | 0.40 |
| Gender (Female) |  | 0.03 | 0.55 | 0.06 |
| Group (Control) |  | -0.177 | 0.51 | -0.348 |
| *Step 2* |  |  |  |  |
| Constant |  | 0.50 | 2.60 | 0.19 |
| BART |  | 0.01 | 0.02 | 0.26 |
| AISS Intensity |  | -0.01 | 0.07 | -0.22 |
| AISS Novelty |  | -0.04 | 0.08 | -0.48 |
| IGT Score |  | -0.01 | 0.01 | -1.32 |
| BIS Attentional |  | 0.09 | 0.07 | 1.28 |
| BIS Motor |  | -0.06 | 0.08 | -0.71 |
| BIS Non-planning |  | 0.04 | 0.07 | 0.60 |
| SST Commission |  | 0.07 | 0.08 | 0.92 |
| SST Omission |  | 0.01 | 0.02 | 0.41 |
| Group (Control) |  | -0.17 | 0.51 | -0.34 |
| *Step 3* |  |  |  |  |
| Constant |  | 0.42 | 2.59 | 0.16 |
| BART |  | 0.002 | 0.02 | 0.17 |
| AISS Novelty |  | -0.05 | 0.07 | -0.66 |
| IGT Score |  | -0.01 | 0.01 | -1.33 |
| BIS Attentional |  | 0.09 | 0.07 | 1.29 |
| BIS Motor |  | -0.06 | 0.08 | -0.73 |
| BIS Non-planning |  | 0.04 | 0.06 | 0.68 |
| SST Commission |  | 0.08 | 0.08 | 1.05 |
| SST Omission |  | 0.01 | 0.02 | 0.02 |
| Group (Control) |  | 0.17 | 0.49 | -0.35 |
| *Step 4* |  |  |  |  |
| Constant |  | 0.38 | 2.59 | 0.15 |
| AISS Novelty |  | 0.05 | 0.07 | -0.66 |
| IGT Score |  | -0.01 | 0.01 | -1.30 |
| BIS Attentional |  | 0.09 | 0.07 | 1.34 |
| BIS Motor |  | -0.06 | 0.08 | -0.79 |
| BIS Non-planning |  | 0.05 | 0.06 | 0.77 |
| SST Commission |  | 0.08 | 0.07 | 1.18 |
| SST Omission |  | 0.01 | 0.02 | 0.40 |
| Group (Control) |  | -0.15 | 0.49 | -0.30 |
| *Step 5* |  |  |  |  |
| Constant |  | 0.47 | 2.60 | 0.18 |
| AISS Novelty |  | -0.57 | 0.07 | -0.80 |
| IGT Score |  | -0.01 | 0.01 | -1.34 |
| BIS Attentional |  | 0.09 | 0.07 | 1.31 |
| BIS Motor |  | -0.06 | 0.08 | -0.79 |
| BIS Non-planning |  | 0.06 | 0.06 | 0.94 |
| SST Commission |  | 0.84 | 0.72 | 1.17 |
| SST Omission |  | 0.01 | 0.02 | 0.37 |
| *Step 6* |  |  |  |  |
| Constant |  | 1.02 | 2.07 | 0.49 |
| AISS Novelty |  | -0.06 | 0.07 | -0.80 |
| IGT Score |  | -0.01 | 0.01 | -1.34 |
| BIS Attentional |  | 0.09 | 0.70 | 1.29 |
| BIS Motor |  | -0.69 | 0.07 | -0.94 |
| BIS Non-planning |  | 0.06 | 0.06 | 0.94 |
| SST Commission |  | 0.07 | 0.06 | 1.18 |
| *Step 7* |  |  |  |  |
| Constant |  | 0.07 | 1.59 | 0.04 |
| IGT Score |  | -0.01 | 0.01 | -1.36 |
| BIS Attentional |  | 0.08 | 0.07 | 1.21 |
| BIS Motor |  | -0.08 | 0.07 | -1.18 |
| BIS Non-planning |  | 0.05 | 0.06 | 0.83 |
| SST Commission |  | 0.06 | 0.06 | 1.07 |
| *Step 8* |  |  |  |  |
| Constant |  | 0.70 | 1.58 | 0.45 |
| IGT Score✝ |  | -0.11 | 0.01 | 1.91 |
| BIS Attentional✝ |  | 0.11 | 0.63 | 1.75 |
| BIS Motor |  | -0.78 | 0.07 | -1.12 |
| SST Commission |  | 0.05 | 0.06 | 0.82 |
| *Step 9* |  |  |  |  |
| Constant |  | 0.91 | 1.56 | 0.58 |
| IGT Score✝ |  | -0.01 | 0.01 | 1.95 |
| BIS Attentional |  | 0.10 | 0.06 | 1.60 |
| BIS Motor |  | -0.07 | 0.07 | -1.04 |
| *Step 10* |  |  |  |  |
| Constant |  | -0.22 | 1.02 | -0.21 |
| IGT Score✝ |  | -0.01 | 0.01 | -1.87 |
| BIS Attentional |  | 0.07 | 0.06 | 1.18 |

BART, Balloon Analogue Risk Task; AISS, Arnett Inventory of Sensation Seeking; IGT, Iowa Gambling Task; BIS, Barratt Impulsiveness Scale; SST, Stop Signal Task. ✝ *p* <.07

**Table 9.** Summary of negative binomial regression models of variables related to prior alcohol use behaviour.

| Variable |  | β | S.E. β | z |
| --- | --- | --- | --- | --- |
| *Step 1* |  |  |  |  |
| Contestant |  | 0.78 | 0.69 | 1.13 |
| AUDIT |  | 0.01 | 0.07 | 0.87 |
| Units/week |  | -0.002 | 0.02 | -0.11 |
| BDS |  | 0.37 | 0.07 | 0.53 |
| Gender (Female) |  | 0.40 | 0.47 | 0.85 |
| Group (Control) |  | -0.52 | 0.50 | -1.05 |
| *Step 2* |  |  |  |  |
| Contestant |  | 0.80 | 0.68 | 1.17 |
| AUDIT |  | 0.01 | 0.04 | 0.14 |
| BDS |  | 0.04 | 0.07 | 0.52 |
| Gender |  | 0.39 | 0.47 | 0.84 |
| Group |  | -0.515 | 0.493 | -1.045 |
| *Step 3* |  |  |  |  |
| Contestant |  | 0.77 | 0.62 | 1.25 |
| BDS |  | 0.06 | 0.06 | 0.95 |
| Gender (Female) |  | 0.28 | 0.49 | 0.57 |
| Group (Control) |  | -0.78 | 0.48 | -1.61 |
| *Step 4* |  |  |  |  |
| Contestant |  | 0.96 | 0.52 | 1.86 |
| BDS |  | 0.05 | 0.62 | 0.87 |
| Group (Control)✝ |  | -0.82 | 0.49 | -1.68 |
| *Step 5* |  |  |  |  |
| Contestant |  | 1.28 | 0.30 | 4.33 |
| Group (Control) |  | -0.62 | 0.49 | -1.28 |

AUDIT, Alcohol Use Disorders Identification Test; BDS, Binge Drinking Scale. N.B. Alcohol units/week: self-reported weekly alcohol consumption, in UK units (1 UK unit = 8g pure ethanol). ✝ *p* <.09.

**Table 10.** Summary of the negative binomial regression model with subjective drink enjoyment as a covariate.

| Variable |  | β | S.E. β | z |
| --- | --- | --- | --- | --- |
| *Step 1* |  |  |  |  |
| Constant |  | 0.39 | 0.64 | 0.61 |
| Mean Drink Enjoyment* |  | 0.12 | 0.06 | 2.04 |
| Gender (Female) |  | 0.28 | 0.29 | 0.98 |
| Group (Control) |  | -0.28 | 0.30 | -0.93 |
| *Step 2* |  |  |  |  |
| Constant |  | 0.16 | 0.64 | 0.24 |
| Mean Drink Enjoyment* |  | 0.13 | 0.06 | 2.17 |
| Gender (Female) |  | 0.36 | 0.29 | 1.22 |
| *Step 3* |  |  |  |  |
| Constant |  | 0.55 | 0.58 | 0.94 |
| Mean Drink Enjoyment✝ |  | 0.11 | 0.60 | 1.83 |

✝ *p* <.07.

**Table 11.** Summary of the final negative binomial regression models following backwards elimination.

| Variable |  | β | S.E. β | z |
| --- | --- | --- | --- | --- |
| *Step 1* |  |  |  |  |
| Constant |  | 1.42 | 0.62 | 2.31 |
| HR Reactivity✝ |  | 0.03 | 0.02 | 1.70 |
| HR Recovery** |  | -0.07 | 0.02 | -3.27 |
| SDNN Reactivity** |  | 0.05 | 0.02 | 2.20 |
| SDNN Recovery* |  | -0.06 | 0.03 | -2.48 |
| IGT Score** |  | -0.01 | 0.003 | -2.97 |
| Mean Drink Enjoyment✝ |  | 0.08 | 0.05 | 1.71 |
| Gender (Female) |  | -0.15 | 0.23 | 0.67 |
| Group (Control)** |  | -0.81 | 0.30 | -2.68 |
| *Step 2* |  |  |  |  |
| Constant |  | 1.22 | 0.54 | 2.27 |
| HR Reactivity |  | 0.03 | 0.02 | 1.61 |
| HR Recovery** |  | -0.07 | 0.02 | -3.19 |
| SDNN Reactivity** |  | 0.05 | 0.02 | 3.12 |
| SDNN Recovery* |  | -0.06 | 0.03 | -2.42 |
| IGT Score** |  | -0.01 | 0.003 | -2.88 |
| Mean Drink Enjoyment* |  | 0.09 | 0.05 | 2.08 |
| Group (Control)** |  | -0.74 | 0.28 | -2.64 |
| *Step 3* |  |  |  |  |
| Constant |  | 1.29 | 0.54 | 2.41 |
| HR Recovery** |  | -0.04 | 0.01 | -3.26 |
| SDNN Reactivity** |  | 0.04 | 0.02 | 2.75 |
| SDNN Recovery* |  | -0.05 | 0.03 | -2.15 |
| IGT Score** |  | -0.01 | 0.003 | -3.08 |
| Mean Drink Enjoyment* |  | 0.09 | 0.05 | 2.01 |
| Group (Control)** |  | -0.80 | 0.27 | -2.93 |

HR, heart rate; SDNN, standard deviation of NN intervals; IGT, Iowa Gambling Task. * *p* <.01, ** *p* < .05, ✝ *p* <.09.
